# COVID-19 pandemic and the great impulse to telemedicine: the basis of the WONCA Europe Statement on Telemedicine at the WHO Europe 70th Regional Meeting September 2020

**DOI:** 10.1017/S1463423621000633

**Published:** 2021-12-13

**Authors:** Ferdinando Petrazzuoli, Donata Kurpas, Shlomo Vinker, Valentina Sarkisova, Androulla Eleftheriou, Anna Żakowicz, Diederik Aarendonk, Mehmet Ungan

**Affiliations:** 1 The European Regional Network of the World Organization of Family Doctors (WONCA Europe); 2 European Rural and Isolated Practitioner Association (EURIPA); 3 European General Practice Research Network (EGPRN); 4 Department of Clinical Sciences in Malmö, Centre for Primary Health Care Research, Lund University, Malmö, Sweden; 5 Department of Family Medicine, Wroclaw Medical University, Wroclaw, Poland; 6 Department of Family Medicine Sackler Faculty of Medicine, Tel Aviv University, Israel; 7 European Forum of National Nursing and Midwifery Associations (EFNNMA); 8 Thalassaemia International Federation (TIF); 9 AIDS Healthcare Foundation Europe (AHF EUROPE); 10 European Forum for Primary Care (EFPC); 11 Department of Family Medicine, Ankara University School of Medicine, Ankara, Turkey

**Keywords:** access to care, rural health, telemedicine

## Abstract

Telemedicine is the use of telecommunication and information technologies to support the delivery of healthcare at a distance, guaranteeing patients healthcare by facilitating access where barriers exist; the COVID-19 pandemic has attracted worldwide interest in this field.

The purpose of this paper is to highlight the main pros and cons of telemedicine, which serve as the basis of the WONCA Europe Statement at the WHO Europe 70th Regional Meeting on 14 September 2020.

Pros of telemedicine include virtual healthcare at home, where patients receive support in certain conditions without leaving their houses. During a pandemic, it can be adopted to limit physical human interaction. Unfortunately, it can negatively affect the quality of the doctor–patient relationship, the quality of the physical examination, and the quality of care. Telemedicine requires effective infrastructure and robust investments to be feasible and effective.

## Introduction

Telemedicine received a big boost from the COVID-19 pandemic where it was adopted to limit physical human interaction (Ortega *et al.*, 2020). It also proved useful in particular to help conserve personal protective equipment and provide isolated COVID-positive patients’ connection to friends and family (Wosik *et al.*, 2020). In that respect, a recent cluster-randomized trial showed that telemedicine can also be useful in the treatment of depression and anxiety in routine primary care (Berryhill *et al.*, 2019; Balestrieri *et al.*, 2020). During the COVID-19 pandemic, telemedicine solutions such as teleconsultations previously used in epidemics such as Ebola and SARS gained greater visibility (Bokolo, 2020; Greenhalgh *et al.*, 2020; Hollander and Carr, 2020; Joy *et al.*, 2020; Baudier *et al.*, 2021).

Telemedicine supplies the main aim of reducing the level of contact among people to prevent cross-contamination and avoid the spreading of the virus. Nevertheless, the goal of teleconsultation is also to continue providing patients with medical support, whether infected by COVID-19 or not. (Campbell, 2020; Richard, 2020; Rockwell and Gilroy, 2020; Baudier *et al.*, 2021).

Family doctors/general practitioners (FDs/GPs) were first in and probably will be last out of the pandemic, and as such telemedicine has an important role in primary care.

Telemedicine is the use of telecommunication and information technologies to support the delivery of healthcare at a distance. Telehealth has a broader definition, encompassing telemedicine’s clinical care and tele-education for research, disaster planning, and primary healthcare at geographically distant and poorer areas (Eisenstein *et al.*, 2020). Telemedicine and telehealth are terms commonly used today and are considered synonymous or complementary. (Pereira *et al.*, 2012).

The World Health Organization (WHO) describes telemedicine as follows: telemedicine involves the use of telecommunications and virtual technologies to provide healthcare outside traditional health facilities (World Health Organization, 2017). Examples of telemedicine include virtual healthcare at home, where patients such as the chronically ill or the elderly can receive remote support in certain treatments at their house, and it also facilitates communication between healthcare professionals in isolated and remote environments. Training can also sometimes be achieved through telemedicine programs or associated technologies such as eHealth, which use computers and the Internet (Pereira *et al.*, 2012).

Telemedicine in its various forms has the great potential to guarantee patients healthcare by facilitating access where barriers exist (Ray *et al.,* 2015; Reed *et al.*, 2019). Getting the best diagnosis and treatment is a right of all people regardless of where they live, and telemedicine may come to the rescue above all to populations living in remote areas, for example in high mountains, on islands, or in areas with poor medical coverage, to compatriots living abroad or who are abroad for travel, to sailors or oil platforms workers, and to all people who, for physical, family, or work reasons, cannot move from their city of residence (Johansson, Lindberg, & Söderberg, 2016). ‘No one left behind’ is a motto by the WHO, and telemedicine may help the primary care team reach patients, especially in this pandemic (World Health Organization, 2017).

Digital thermometers, blood pressure, blood glucose, blood oxygen, and heart rate monitoring systems are noninvasive devices commonly used at home. Additionally, there are more sophisticated (and expensive) remote aids, for example, the remote stethoscope that received impetus from the COVID-19 pandemic (Vest *et al.*, 2016; Amster *et al.*, 2020; Vidal-Alaball *et al.*, 2020).

In this article, we will highlight the potential benefits of the implementation of telemedicine in primary care, especially in rural settings; as well as the disadvantages. The opportunities and threats identified provided the basis for the WONCA Europe Statement at the 70th session of the WHO Regional Committee for Europe on 14 September 2020 (Supplementary material). The ongoing pandemic has highlighted the benefits and limitations of telemedicine. The widespread lack of unified guidelines and the heterogeneity of primary care (especially in rural areas) were the main impetus for this position paper.

### Methods for the statement/position paper

The statement was the product of WONCA Europe which invited its rural Network EURIPA to start developing a statement on telemedicine with a focus on rural settings. After exhaustive discussion within European Rural and Isolated Practitioners Association (EURIPA), the statement was approved by the WONCA Europe executive and then endorsed by other partner organizations of the WHO, namely the European Forum of National Nursing and Midwifery Associations (EFNNMA), the Thalassaemia International Federation (TIF), the AIDS Healthcare Foundation Europe (AHF EUROPE), and the European Forum for Primary Care (EFPC).

## Potential benefits for patients and healthcare staff

Video or telephone visits can give patients real-time access to a doctor without the need to travel. It has been estimated that for a 20-min medical office visit, patients in the United States spend an average of 2 h, including travel and waiting times (Ray *et al.*, 2015; Dorsey & Topol, 2016).

According to Vidal Alabal *et al* (Vidal-Alaball *et al.*, 2020), adoption of telemedicine and virtual software platforms aids in the following ways: decreases the time required to get a diagnosis and initiate treatment, stabilizes, or quarantines a patient; facilitates close follow-up with patients who can be monitored from their home to avoid crowding of health facilities; reduces movement of people, minimizes the risk of intra-clinic infection; supports coordination of medical resources utilized in distant locations; aids in informing the general public; and lastly, prevents contagion, particularly for medical practitioners, the key assets of the health system.

### Disadvantages of telemedicine

The disadvantages of telemedicine include the quality of the doctor–patient relationship, the quality of the physical examination, and the quality of care (Totten *et al.*, 2016; Randhawa *et al.*, 2018). By their very nature, telemedicine visits have the potential to undermine the quality of doctor–patient interaction in several ways. First, the ability to build patient trust is more difficult remotely than in person (Nittari *et al.*, 2020). The problem is even greater if the remote consultation is with clinicians with whom the patient has not already established a relationship (Dorsey & Topol, 2016). The quality of a remote ‘physical’ consultation is clearly inferior to the quality of an actual physical examination. Initially, telemedicine applications focused on conditions when the physical examination is not essential for the encounter (e.g., tele-radiology) or were based on visual assessment (e.g., dermatology) (Nittari *et al.*, 2020). The limitations of a remote ‘physical’ examination can be substantial, for example, the inability to palpate an acute abdomen (e.g., appendicitis), or the fovea sign in patients with congestive heart failure (Dorsey & Topol, 2016).

An important disadvantage that should be the focus of future research is the increase in health service demand due to the easier access. This process may increase competition over resources and increase inequity between more and less technological populations (Dorsey & Topol, 2016). Another problem that may be seen is a shift to consultations at earlier stages of complaints (that may be self-limited) leading to over diagnosis and overtreatment (Dorsey & Topol, 2016).

## Telemedicine as an opportunity in primary care, to reduce the disruption of care for chronic condition during a pandemic

During this COVID-19 pandemic, chronic elderly patients are more at risk and have more difficulties accessing care and treatment. According to a recent study, a substantial increase in the number of avoidable cancer deaths in England are to be expected as a result of diagnostic delays due to the COVID-19 pandemic (Maringe *et al.*, 2020).

The care of common chronic diseases such as hypertension has also been seriously affected. There has been disruption of medical supply chains, cancelation of clinics during local lockdowns, and the social distancing protocols have limited the access to care (Skeete *et al.,* 2020).

Primary care doctors should be aware that people are scared of going to hospitals and this may delay the diagnosis and treatment. The enormous efforts to deal with COVID-19 have also disrupted the regular care often required by patients with non-communicable diseases. The excess deaths from the disruption caused by COVID-19 might make any gains against the virus a pyrrhic victory. Primary care response to these issues should include advocacy, prevention, monitoring, and treatment (Lancet, The 2020; McCloskey *et al.*, 2021).

## Factors that impact the widespread adoption of telemedicine

Factors that impact the widespread adoption of telemedicine can be divided into social, organizational, and technological. Social factors include patients’ age, educational level, and social class. Organizational factors include availability of funding, inadequate training, and workflow integration. Technological factors include data privacy and security protection, broadband access, Wi-Fi quality, and availability of IT infrastructure. Other important factors are licensure requirements, health insurance and reimbursement policies, and lack of regulation and advocacy (Hollander & Carr, 2020; Pappot, Taarnhøj, & Pappot, 2020). Telemedicine was shown to be helpful in previous outbreaks, including former coronavirus outbreaks such as the SARS-CoV (severe acute respiratory syndrome-associated coronavirus) and MERS-CoV (Middle East respiratory syndrome coronavirus), or PHEICs (Public Health Emergency of International Concern) related to Ebola and Zika viruses but it is with the COVID-19 pandemic that it has received a worldwide stimulus (Ohannessian, Duong, & Odone, 2020). Law and regulations must adapt to this unexpected change: in France, for example, the Ministry of Health signed a decree on 9 March 2020, allowing the reimbursement of video teleconsultations and tele-expertise by the National Health Insurance (NHI), for patients with COVID-19 symptoms and those confirmed with COVID-19 throughout the country, without the need to know the patient beforehand (Ohannessian *et al.*, 2020).

## Concerns about privacy

Ethics, data confidentiality, informed consent, and medical security are essential components of this new technology, and ensuring transparency is a part of this technological digital strategy option (Langarizadeh, Moghbeli, & Aliabadi, 2017; World Health Organization, 2017; Kaplan, 2020).

In adopting telemedicine and virtual software platforms, physicians must obtain patient consent for online consultation (mostly automated in virtual software platforms privacy statement shown to users during installation of the software), document the type of consultation (e.g., synchronous or asynchronous), location of patient and physician, confirm identity, and document medical service performed (date, time, and duration) (Langarizadeh *et al.*, 2017).

This progressive change in healthcare may open new doors in legal, ethical, and regulatory issues and have a great impact on decision policy-making by health authorities (Nittari *et al.*, 2020).

While misdiagnosis can occur both in face-to-face and virtual interactions, the former has a standard, detailed mechanism from patient complaints to investigation and compliance standards. According to the literature, the risk of misdiagnosis appears to be greater in telemedicine, while the legal statutory clauses are not standardized or universal (Nittari *et al.*, 2020). This often leads to varying standards and coverage offered by service providers and may lead to a decrease in the quality of handling ethical and legal concerns. Potential issues of negligence and malpractice will also have an impact on telemedicine.

## Opportunities and threats in rural settings

Telemedicine has shown to be especially useful in underserved communities where a shortage or absence of adequate clinical care exists, such as in rural and remote areas (Lancet, The 2020; Nittari *et al.*, 2020).

The situation has radically changed over the recent years: a survey of attitudes to eHealth of doctors and nurses in rural general practices in the United Kingdom carried out in 2005 in United Kingdom, showed that although primary healthcare professionals recognized the general benefits of eHealth, the real uptake was still low (Richards *et al.*, 2005). Preliminary data of an ongoing survey by the WONCA Europe network of the rural GPs, the EURIPA, show that telemedicine is valued by rural primary care clinicians, with only 5 % of the respondents reporting that they never used remote consultations during the pandemic. Although most of the remote consultations were performed by phone (about 65%), video consultations were used by approximately 25% on the respondents. Telemedicine appears also to be appreciated by rural patients, with only less than 10 % of the patients responding they ‘did not like it’, supporting this to be an effective alternative to face-to-face consultations (Kern *et al.*, 2020).

These data are useful, as no major research has been carried out on the issue of doctors’ and patients’ satisfaction (Totten *et al.*, 2016; Tuckson, Edmunds, & Hodgkins, 2017; Reed *et al.*, 2019; Reed *et al.*, 2020).

As the healthcare system becomes increasingly virtual, there is a risk of widening disparity, with marginalized populations (having worse health outcomes at baseline) and limited access to the resources necessary for effective telemedicine (Ortega *et al.*, 2020).

One of the potential dangers of telemedicine is that some countries may use it as a tool to cut health services, especially in rural areas. Telemedicine should be an alternative, for clinicians to determine, with their patient, the best way to deliver services (Table 1).

**Table 1.** Key points of the WONCA Europe Statement on Telemedicine at the WHO Europe 70th Regional Meeting September 2020 (supplementary material)

Having access to the highest quality of care is a right of all people regardless of where they live. Telemedicine presents an opportunity for populations living in rural and remote areas. Telemedicine should include intra- and interdisciplinary care involving all health and social care professionals and local communities (patients and their informal caregivers). Telemedicine is a tool to support the delivery of high-quality health services and should not be used to cut services. The non- state actors (NSAs) supporting this statement urge the WHO member states to action to progress the use of telemedicine as a tool to support the practice of family medicine for the benefit of its patients: to undertake health technology assessments to ensure that the implementation of telemedicine is equitable for all primary care clinicians and their patients across the different geographies and demographics of Europe to engage PHC organizations in collaboration with governments, policy-makers, and other stakeholders to ensure proper implementation and regulation of telemedicine in order to achieve equitable availability and acceptability of care for all people living in Europe. Collaboration is essential to ensure a robust patient family-centered service to ensure that telemedicine is not used as a tool to cut services, especially in rural areas. Telemedicine is another way for a clinician to determine, together with the patient, the best way to deliver services European politicians, governments, and other decision-makers are urged to address the digital divide including the provision of infrastructure such as broadband and mobile coverage in remote and rural areas to address the urgent need for official recognition of telemedicine by heath/social care and the development of guidelines on its use, but to minimize bureaucracy that could hinder the development and the implementation of formal and informal telemedicine to ensure that new strategies, policies, and models for service delivery are effectively rural proofed to ensure equity of opportunity for rural remote populations and their primary care clinicians As the NSAs in PHC, we wish to strongly emphasize that the use of telemedicine as a tool should support delivery of primary healthcare services equitably in all areas across Europe.

## Conclusion

Telemedicine has many advantages and can be a great opportunity in the care of disadvantaged populations. It has the potential to break down inequalities, but care must be taken that it not be used as a tool to cut or replace services. We need to regulate this activity, but we also need to minimise bureaucracy that could hinder the development and the implementation of formal and informal telemedicine. We are optimistic in the effective use of telemedicine in the near future, but there are still aspects that are not well developed or with obvious shortcomings.

## Supplementary material

For supplementary material accompanying this paper visit http://dx.doi.org/10.1017/S1463423621000633. click here to view supplementary material
