# Supplementary material for: COVID-19 pandemic and the great impulse to telemedicine: the basis of the WONCA Europe Statement on Telemedicine at the WHO Europe 70th Regional Meeting September 2020
Source: Prim Health Care Res Dev. 2021 Dec 13;22:e80. doi: 10.1017/S1463423621000633 (PMC8695945; doi:10.1017/S1463423621000633)
Supplement: Supplementary file 1 [file S1463423621000633sup001.pdf]

DIGITAL HEALTH & TELEMEDICINE IN PRIMARY CARE ADDRESSING ALSO INEQUITY IN RURAL HEALTH

STATEMENT ON PROVISIONAL AGENDA ITEM 2: lessons learned from the COVID-19 pandemic.

In response to the Covid-19 pandemic modern information and communication technologies have been rapidly introduced to deliver health services. Telemedicine in primary care includes triage, sharing of images and remote consultations using audio and/or video technology. Telemedicine is effective, feasible and relatively affordable even in low middle income countries, provided there is sufficient internet access in rural/remote areas.
A recent survey by the WONCA Europe network of the rural GPs, the European Rural and Isolated Practitioners Association, shows that telemedicine is valued by rural primary care clinicians and appears to be appreciated by rural patients as well. During the pandemic it has been an effective alternative to most face to face consultations.
As Europe moves out of the lockdown, telemedicine's role in the 'new' normality should be critically appraised to avoid unforeseen consequences. Telemedicine is a tool to support the delivery of health services and support the relationship between a GP and patient. Culture, language, technological and health literacy need to be taken into account. Patient and community involvement are needed to develop future services.
Rural areas across Europe are characterised by older populations, low density of population, , remoteness from services and are frequently lacking infrastructure to support the new information technologies. In normal times telemedicine can provide an opportunity to overcome some accessibility issues in rural and remote areas. It cannot be seen as a replacement for traditional primary care in challenging areas such as rural and remote areas. Remote on-line GP services can also pose a threat to traditional family medicine and could destabilise it.

WONCA Europe and its rural network of rural GPs states that:

> *Having access to the highest quality of care is a right of all people regardless of where they live. Telemedicine presents an opportunity for populations living in rural and remote areas. Telemedicine should include intra- and interdisciplinary care involving all health and social care professionals and local communities (patients and their informal caregivers). Telemedicine is a tool to support the delivery of high-quality health services and should not be used to cut services.*

The NSAs supporting this statement urge the WHO Member States to action to progress the use of telemedicine as a tool to support the practice of family medicine for the benefit of its patients:

- To undertake health technology assessments to ensure that the implementation of telemedicine is equitable for all primary care clinicians and their patients across the different geographies and demographies of Europe.

- To engage PHC organisations in collaboration with governments, policy-makers, and other stake-

holders to ensure proper implementation and regulation of telemedicine in order to achieve equitable availability and acceptability of care for all people living in Europe. Collaboration is essential to ensure a robust patient family centred service.

- To ensure that telemedicine is not used as a tool to cut services, especially in rural areas. Telemedicine is another way for a clinician to determine, together with the patient, the best way to deliver services.

- European politicians, governments and other decision makers are urged to address the Digital Divide including the provision of infrastructure such as broad band and mobile coverage in remote and rural areas.

- To address the urgent need for official recognition of Telemedicine by heath/social care and the development of guidelines on its use, but to minimise bureaucracy that could hinder the development and the implementation of formal and informal telemedicine.

- To ensure that new strategies, policies and models for service delivery are effectively rural proofed to ensure equity of opportunity for rural remote populations and their primary care clinicians.

As the NSAs in PHC we wish to strongly emphasise that the use of telemedicine as a tool should support delivery of primary health care services equitably in all areas across Europe

Non State Actors (NSA) & NGOs supporting this statement

- WONCA Europe: The European regional network of the World Organization of Family Doctors (WONCA) represents more than 150,000 family doctors through 48 Member Organisations across Europe.

- EFNNMA: European Forum of National Nursing and Midwifery Associations

- Thalassaemia International Federation

- AHF Europe

- European Forum for Primary Care

Ferdinando Petrazzuoli MD, PhD
WONCA Europe network of rural GPs
Scientific Board chair

Ferdinando Petrazzuoli

Professor Mehmet Ungan
Wonca Europe President
